# What can artificial intelligence teach us about the molecular mechanisms underlying disease?

**DOI:** 10.1007/s00259-019-04370-z

**Published:** 2019-06-12

**Authors:** Gary J. R. Cook, Vicky Goh

**Affiliations:** 1 Cancer Imaging Department, School of Biomedical Engineering and Imaging Sciences, King’s College London, London, SE1 7EH UK; 2 King’s College London & Guy’s and St Thomas’ PET Centre, St Thomas’ Hospital, London, UK; 3 Radiology Department, Guy’s and St Thomas’ Hospitals NHS Trust, London, UK

**Keywords:** Radiomics, Machine learning, deep learning, Artificial intelligence, Molecular imaging

## Abstract

While molecular imaging with positron emission tomography or single-photon emission computed tomography already reports on tumour molecular mechanisms on a macroscopic scale, there is increasing evidence that there are multiple additional features within medical images that can further improve tumour characterization, treatment prediction and prognostication. Early reports have already revealed the power of radiomics to personalize and improve patient management and outcomes. What remains unclear is how these additional metrics relate to underlying molecular mechanisms of disease. Furthermore, the ability to deal with increasingly large amounts of data from medical images and beyond in a rapid, reproducible and transparent manner is essential for future clinical practice. Here, artificial intelligence (AI) may have an impact. AI encompasses a broad range of ‘intelligent’ functions performed by computers, including language processing, knowledge representation, problem solving and planning. While rule-based algorithms, e.g. computer-aided diagnosis, have been in use for medical imaging since the 1990s, the resurgent interest in AI is related to improvements in computing power and advances in machine learning (ML). In this review we consider why molecular and cellular processes are of interest and which processes have already been exposed to AI and ML methods as reported in the literature. Non-small-cell lung cancer is used as an exemplar and the focus of this review as the most common tumour type in which AI and ML approaches have been tested and to illustrate some of the concepts.

## Introduction

Positron emission tomography (PET) and single photon emission computed tomography (SPECT) already provide macroscopic information on aberrant molecular pathways and altered cellular biology in several diseases. This is highly relevant in cancer, since most of the hallmarks of cancer [1] can now be imaged and quantified using PET and SPECT radiopharmaceuticals. Examples include cellular processes that have histopathological correlates such as proliferation, apoptosis and antigen or receptor expression, as well as altered molecular pathways, including the processes that contribute to tumour metabolism, hypoxia and angiogenesis. Measurement of these processes is crucial to the understanding of individual tumour phenotypes, helping us understand how a tumour will behave with regard to local invasion or metastatic potential, understand the relationship and interaction of tumour cells with the microenvironment, and predict and monitor response or resistance to therapy. While the reference standard in clinical practice remains histopathology and immunohistochemistry (IHC), there are potential advantages of using in vivo imaging.

Molecular profiling is an increasingly common approach to the stratification of patients for targeted therapy. Imaging can augment histopathological and IHC measures based on tissue biopsies in that a whole tumour and its microenvironment can be noninvasively interrogated, so that heterogeneous tumours are not subject to histopathological sampling error. Whole-body imaging also captures the heterogeneity of underlying molecular and cellular processes between different tumours in the same patient.

Radiomics is a developing area of imaging that involves the extraction of multiple features from medical images that, with bioinformatic approaches, can be used to provide additional information that may predict underlying tumour biology and behaviour [2–4]. This relies on the hypothesis that individual voxel values and their spatial distribution within a tumour are influenced by, and may represent or correlate with, underlying biological processes causing differences in attenuation (CT), tissue relaxation (MRI) or tracer uptake (SPECT and PET) [5–10]. Radiomic signatures can be used alone or with other patient-specific data to improve tumour phenotyping, treatment response prediction and prognosis, noninvasively by recognizing patterns on a global or locoregional scale [11].

While a radiologist or nuclear medicine physician relies on the recognition of a handful of semantic features to detect and describe tumours, thousands of agnostic features can potentially be extracted from medical images. This complexity within medical images extending beyond the scope of the human brain is amenable to analysis by artificial intelligence (AI), and in particular machine learning (ML), approaches, that will reveal the additional information that the images may hold. ML approaches enable computers to create predictive algorithms and to learn without any explicit programmed rules, i.e. learning through experience from new datasets. Deep-learning approaches are a subset of ML based on artificial neural networks that mimic how the nervous system processes information.

Indeed, radiomics has progressed from direct selection of predefined features that can be used alone or in combination as inputs into ML classifiers, to obtaining indirect learned features without a priori definition using deep-learning data-driven methodology. The latter methods may be more generalizable, as they do not require preprocessing, for example segmentation [12–14], complex patterns may be ‘discovered’ in an objective and automated way, and multimodality and multiparametric scales may therefore be more easily accommodated. They may also overcome statistical issues of overfitting, collinearity and redundancy of features, although these advantages need to be more clearly determined in molecular imaging techniques and are dependent on the size of the training dataset, molecular imaging datasets usually being relatively small [15, 16].

The scale of data required for ML approaches is generally large. For retrospective analysis, the use of pre-existing data can bring challenges, especially if the data were acquired using different imaging protocols that may have introduced additional variability between the datasets. Data sharing, data protection, ethical and patient consent issues are common public concerns; data curation can also be problematic. For the practising clinician, there are also issues related to the ‘black box’ nature of many deep-learning approaches. With the lack of regulation and standards, such approaches can be opaque. Furthermore, it may not be possible to understand how patterns are discovered because of our limited or absent theoretical understanding of what ML ‘sees’.

The question we try to address in this review is whether the use of AI, with a focus on PET and SPECT, can provide incremental insights into cellular and molecular mechanisms in cancer. We consider why molecular and cellular processes are of interest and which processes have already been exposed to AI and ML methods as reported in the literature. We use non-small-cell lung cancer (NSCLC) as an exemplar since it is the most common tumour type and is the tumour type in which AI and ML approaches have been tested.

## Why is knowledge of underlying biological and molecular mechanisms of interest?

Biological and molecular mechanisms provide insight into the tumour phenotype and predicted behaviour. On a simple level, it is recognized that malignant tumours show heterogeneity of molecular and cellular features, including cellular density and proliferation, necrosis, fibrosis, metabolism, hypoxia, angiogenesis and receptor expression, factors that have been independently associated with a poor treatment response and more aggressive tumour behaviour [1]. This variation, defined by histopathological appearance, may in turn reflect the degree of genetic clonal variation. These biological processes can be crudely determined using functional and molecular imaging methods on a global scale, and there is some evidence that several of these adverse biological features may be reflected in medical images. However, it is likely that measurement of their heterogeneous expression will require more sophisticated analysis to which radiomic and AI methodology could contribute [6, 11–13].

The interest in radiomics and the use of AI to extract additional information from medical images has been accelerated by the knowledge that there is intra- and intertumoral genetic heterogeneity within and between patients and that the genetic profile may change with time and treatment. Genetic heterogeneity within a tumour is not only associated with morphological heterogeneity of the tumour cell nuclei, but also with a poorer prognosis [17]. As a consequence of therapy, heterogeneous tumours are able to more readily adapt to anticancer treatment, leading to resistance and worse treatment outcomes [18–20]. Clonal expansion may not only confer resistance to treatment but may increase drug target heterogeneity and other adverse factors such as metastatic potential.

Increasingly, molecular profiling of individual tumours is used to direct treatment decisions. A common example of this approach is the measurement of *EGFR* gene mutations to direct certain tyrosine kinase inhibitor (TKI) therapeutics that show improved efficacy in mutated tumours [21]. Again, if imaging methods could predict mutational status on a locoregional basis and between different metastatic sites, they could complement standard tissue molecular profiling or replace it in some circumstances, particularly when serial measurements during treatment are required.

As an example, the recent advances in the control of advanced and metastatic cancers, such as NSCLC and melanoma that previously had a dismal prognosis, with immune checkpoint inhibitors is another area in which measurement of underlying molecular and cellular characteristics by imaging techniques could contribute to treatment decisions. Currently drugs that target programmed cell death-1 and its ligand (PD-1, PD-L1) are often selected on the basis of IHC measurement of PD-L1 expression in biopsy material. It is recognized that some patients respond well despite negative PD-L1 expression measured on IHC, and that PD-L1 expression is heterogeneous, suggesting inaccuracies and sampling errors in measurement [22, 23]. Imaging has the potential to reveal global, locoregional and metastatic characteristics associated with PD-L1 expression either directly or by radiomic analysis [24, 25].

With the increasing number of novel PET and SPECT tracers for exploring different aspects of tumour biology, and the more routine use of hybrid multimodality imaging providing multiparametric measurements, increasingly sophisticated methods of analysis, such as ML and AI, will be required to cope with the increasingly large amounts of data and to uncover the radiomic information ‘hidden’ within. In addition, it is possible that integrating radiomic data with genomic and pathological data may further enhance tumour characterization but will also require methods that can interrogate very large datasets [2, 11, 18, 26].

## What molecular and cellular biological data can be measured or inferred from imaging?

How does molecular imaging contribute currently to the pathological characterization and grading of tumours, as well as other standard histopathological features such as cellular density? In a preclinical context, the spatial distribution of ^18^F-fluorodeoxyglucose (FDG) has been shown to reflect the spatial distribution of cellular density, stromal tissue and necrosis in a head and neck cancer murine model [9]. In hepatoma and pancreatic murine tumour models, FDG spatial heterogeneity has been reported to be associated with the distribution of glucose transporters and hexokinase [7, 8]. In orthotopic breast cancer models, a correlation was found between various radiomic texture features describing the spatial distribution of FDG activity in autoradiographic images and the spatial distribution or density of cells determined on histopathological staining [5]. However, PET images of lower spatial resolution than autoradiography only coarsely captured tumour cellular heterogeneity.

Radiomic signatures from FDG PET images in humans have been reported to differentiate NSCLC subtypes [27], breast cancer IHC factors, including HER2 expression [28] and triple-negative status [29], and benign from malignant peripheral nerve sheath tumours in patients with neurofibromatosis-1 [30], as well as other varieties of bone and soft tissue lesions [31]. Cellular proliferation, as measured by ^18^F-fluorothymidine PET, has been linked to gene expression patterns in a murine pancreatic tumour model [7] and heterogeneity of uptake has been reported to be a potential predictive and response marker in patients with breast cancer treated with chemotherapy [32]. Radiomic approaches have also been used to explore the relationships between regional FDG PET and MRI features from combined PET/MRI showing correlations with microvascular density and expression of vascular endothelial growth factor in renal cell carcinoma, with the highest correlations when combining PET and MRI radiomic features [10].

Intratumoral heterogeneity of a therapeutic target can determine the treatment response to radionuclide therapy where the bystander effect is required to kill neighbouring cells that do not express the target. It has been shown in preclinical colon tumour models that express carcinoembryonic antigen (CEA) that treatment response to ^131^I-labelled anti-CEA antibody depends on the vascular supply and CEA distribution [33]. Heterogeneity of CEA expression has been determined microscopically in different tumour models using multifluorescence, but differences between tumour models can also be determined on a more macroscopic scale using texture analysis of ^125^I-A5B7 anti-CEA SPECT imaging [34].

**Fig. 1:**
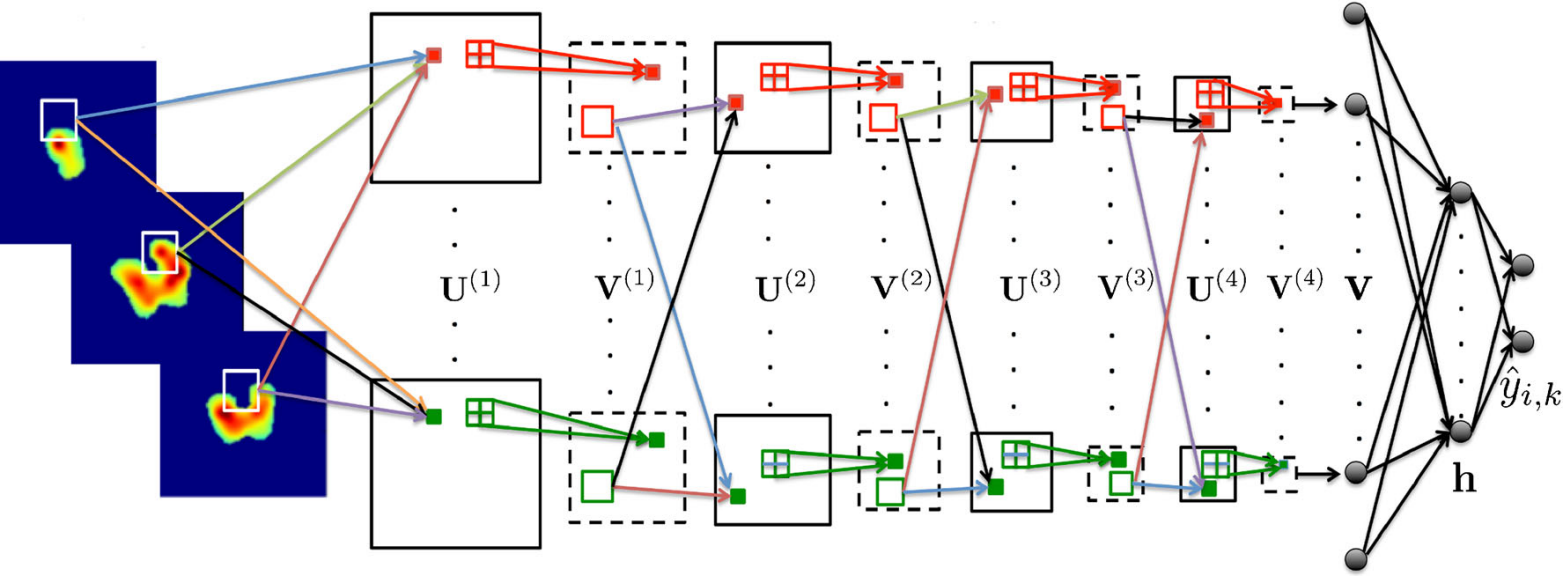
Convolutional neural network architecture for oesophageal cancer ^18^F-FDG PET data in a vector composed from four convolutional (U) and four max-pooling (V) layers. Differently coloured arrows in the first convolutional layer represent different learnable weight matrices. Coloured squares in the feature maps represent elements that include local spatial information from the previous layer. In the max-pooling layers, 2 × 2 element windows represent non-overlapping grids from which the maximum element to down-sample the feature maps are chosen (*h* hidden layer, *y*_*I*_ responder, *y*_*k*_ nonresponder). From Ypsilantis et al. [38]

Several studies using FDG PET have shown relationships between radiomic signatures and treatment response and/or prognosis in a variety of tumours including oesophageal, head and neck and cervical cancers, amongst others. These studies have been well reviewed elsewhere [35, 36], and we summarize the literature on lung cancer below. These studies usually showed incremental benefit over the use of standard parameters, such as standardized uptake values (SUVs), but generally used a wide variety of radiomic features and methodologies, but uncommonly advanced ML or AI techniques. More sophisticated approaches have been reported for predicting treatment response or failure. For example, Vallières et al. used 1,615 radiomic features from FDG PET and CT images to predict treatment failure in head and neck cancer [37]. Prediction models combining radiomic and clinical variables were constructed using random forests (RF) and imbalance adjustment methods and tested on validation datasets. We have reported a comparison of an approach using several statistical and texture parameters with a convolutional neural network (CNN) method on FDG PET scans in patients with oesophageal cancer before treatment. The CNN method outperformed other methods and predicted histopathological nonresponders to neoadjuvant chemotherapy with 81% sensitivity and 82% specificity (Fig. 1) [38].

Whilst there are several reports of associations between mutational status and FDG PET radiomics in lung cancer (see below), some associations have also been reported in colorectal cancer [39–41]. *KRAS*, *TP53* and *APC* mutations were associated with FDG PET SUV parameters and specific radiomic features in these studies. *KRAS* mutation, in particular, is of clinical importance in predicting a poor response to *EGFR*-targeting therapies.

## NSCLC: defining molecular mechanisms from imaging

Attempts have been made to correlate the underlying histological and biological features of NSCLC with radiomic features from FDG PET imaging. Correlations have been observed between histopathological mean cell density and lacunarity (large gaps between clusters of cells) and standard FDG parameters, including SUVmean and total lesion glycolysis, first-order statistical features, including kurtosis and skewness, and FDG lacunarity [42]. Another study has shown associations between a number of texture parameters and NSCLC clinical stage and Ki67 IHC analysis of proliferation using *k*-nearest neighbours and support vector machine (SVM) methods [43]. Differentiation of histopathological tumour subtypes (squamous cell carcinoma and adenocarcinoma) using texture and colour features derived from FDG PET images using a SVM algorithm have also been reported with an area under the receiver operating characteristic curve of 0.89 [27].

Rather than analysing primary tumour FDG PET data, some benefit has been found in analysing lymph node data. Radiomic descriptors derived from metastatic lymph nodes from FDG PET images in patients with NSCLC using a least absolute shrinkage and selection operator (LASSO) method have been found to be more strongly associated with overall survival than when extracted from primary tumour data [44]. Another study employed deep learning (CNN) and ML (RF, SVM, adaptive boosting and artificial neural network). The four ML methods separately used 13 standard diagnostic features (e.g. SUV, tumour size) and 82 textural features to classify mediastinal lymph nodes on FDG PET images in patients with NSCLC [45]. The accuracy of CNN was 86% and was not significantly different from those of the best ML methods that used standard diagnostic features or the combination of diagnostic and textural features. CNN was more accurate than ML methods that used textural features alone, possibly because the lymph nodes were relatively small, which may be a limitation in the calculation of textural features. The sensitivity achieved by radiologists was inferior (73% vs. 84%) but the specificity was better (90% vs. 88%) than those obtained with the CNN method. The advantages of the CNN method, including no need for segmentation or feature definition, were highlighted.

Genetic mutations, including *EGFR* mutations and anaplastic lymphoma kinase (*ALK*) gene rearrangements, that are associated with improved response to certain TKIs, are associated with image features derived from FDG PET in NSCLC [46]. With *EGFR*, findings are conflicting, with some studies predominantly showing high FDG uptake in *EGFR*-mutated tumours, reflecting increased glycolysis through AKT signalling [47], and others showing lower uptake [48]. As well as standard features, such as SUVmax, heterogeneity parameters also show associations with *EGFR* mutation status [49, 50] and value in predicting response and survival following treatment with TKIs [51, 52]. Less specific associations between FDG PET radiomics and genetic heterogeneity, but not tumour mutational burden, have been shown in SCLC but not in NSCLC [53]. Other radiogenomic associations between FDG PET parameters and NSCLC genomics have been described. In a radiogenomic study a prognostic metagene signature derived from 25 patients with NSCLC was associated with a multivariate FDG uptake feature derived from principal components analysis, both of which were associated with survival in external and validation cohorts [54]. The radiogenomic profile was associated with altered cell cycle, proliferation, death and self-recognition pathways, and recognized NF-κB protein as a central node within the metagene. A further study by the same group specifically showed that NF-κB protein expression is associated with high FDG uptake with both being related to advanced tumour stage, grade and invasion [55].

Several studies have shown relationships between NSCLC FDG PET radiomic features and treatment response and survival, and have been reviewed elsewhere [56, 57]. They have predominantly shown incremental benefit over using standard parameters, such as SUV, but most did not use more advanced AI methodology to determine radiomic signatures. In one study, using 201 datasets and 43 textural features, the LASSO method was used and identified a single textural feature (SumMean) as an independent predictor of overall survival in large tumours treated with chemoradiotherapy [58]. In a further study of 358 datasets and 665 radiomic features, a similar LASSO methodology was used to derive predictive feature vectors that were tested on an independent validation set and predicted a 14-month survival difference [59].

## Future perspectives and conclusions

There is no doubt that molecular imaging already provides clinically useful data regarding tumour molecular and biological phenotypes. To date, this has been in a relatively crude manner with measurement of standard global imaging parameters from whole-tumour data, e.g. SUVs. Early reports of more sophisticated methods that extract multiple ‘unseen’ features from images indicate that it is often possible to provide additional information that allows better characterization and treatment stratification, prediction and prognostication. Data on how these techniques relate to the tumour molecular and biological phenotype remains in evolution. Despite a varied approach between laboratories in the early years of radiomics, there is now a more concerted effort to standardize approaches [60].

The resurgent interest in AI methods for medical imaging is related to the increasing complexity of medical imaging data that requires intelligent solutions, and the major advances in graphics processing units and parallel computing approaches has led to ML, and more recently deep learning, approaches being considered for medical purposes. ML, unlike previous rule-based methods, is a powerful and flexible tool that has wide medical imaging applications beyond the assessment of tumour heterogeneity and biology. The use of AI for automated tumour detection, tumour segmentation, tumour biological assessment, automated interpretation of findings and clinical decision support through an integrated pathway may not be so far away from clinical reality. However, AI should not stop us from being clinicians or from thinking critically.

In conclusion, the combination of imaging and clinical and other -omic data will most likely deliver the diagnostic support required for personalized oncology. AI has the ability to extract imaging data rapidly and quantitatively at scale. As well as offering a better understanding of underlying molecular mechanisms of disease, AI has potential to contribute in other areas of imaging and medicine. It is also likely that transfer learning, an approach in which models based on deep learning (that have already been developed for another application and can then be used in a separate or related task, and in which less data are required to build the first layers of a CNN) will accelerate and improve the application of AI to imaging by saving time on training and building of neural networks.
